# Surgery in Dublin

**Published:** 1898-12-31

**Authors:** 


### SURGERY IN DUBLIN.

At the last meeting of the Section of Obstetrics of the Royal Academy of Medicine in Ireland Dr. Alfred J. Smith reported a series of 100 abdominal sections, in continuation of a series of 50 which he had already recorded. Taking the two series together he had performed 150 abdominal sections with only two deaths, or a mortality of hardly 1·3 per cent. The cases included ovarian tumours, separation of adhesions, cure of fibro-myomata, tubal pregnancies, ventral hernia, suppurating tubes, &c., and in nine cases the abdomen had been opened for exploratory purposes. In the discussion an interesting remark was made by Mr. Tobin, of St. Vincent's Hospital, who said that the surgeons of that hospital had all arranged to operate in the same way, and that if any change was to be made in any detail the surgical staff met together and rejected or adopted the proposed change. He said he thought this was an admirable arrangement for all parties. This, perhaps, might be true if the details of surgical technique were less a personal matter than they are. But in existing circumstances we doubt whether any such arrangement is either practicable or good for progress, however well it may speak for the harmony and good fellowship existing among the members of the staff of the hospital where it is in force. Progress is always a matter of individual effort and personal experience—indeed, we might say of personal experiment—and to demand a general acceptance of the new method by the whole staff before any advance was made would, we are afraid, be to hamper all progress, and to take away individual responsibility to an extent which would be by no means counterbalanced by any supposed increase of safety to the patients. Fancy all the surgeons of a London hospital having to make their operative procedures conform to rules passed by the staff in conclave assembled, or an enthusiastic junior exposing his proposed new method to the flouts and sneers of his seniors before he tried it! No, such a thing is not to be thought of.

